# Supplementary material for: Synthetic modified vaccinia Ankara vaccines confer cross-reactive and protective immunity against mpox virus
Source: Commun Med (Lond). 2024 Feb 16;4:19. doi: 10.1038/s43856-024-00443-9 (PMC10873322; doi:10.1038/s43856-024-00443-9)
Supplement: Supplementary file 2 — Supplementary Information [file 43856_2024_443_MOESM2_ESM.pdf]

Synthetic modified vaccinia Ankara vaccines confer cross-reactive and protective immunity against mpox virus

Flavia Chiuppesi; John A. Zaia; Miguel-Angel Gutierrez-Franco; Sandra Ortega-Francisco; Minh Ly; Mindy Kha, Taehyun Kim, Shannon Dempsey; Swagata Kar; Alba Grifoni; Alessandro Sette; Felix Wussow and Don J. Diamond

Supplementary Information

Supplementary Table 1. COH04S1-vaccinated volunteers' characteristics

Characteristic	COH04S1, N = 20	DL1, N = 10	DL2, N = 5	DL3, N = 5	placebo, N = 4
Age[1]	36 (22, 54)	39 (22, 50)	40 (28, 54)	32 (26, 41)	31 (24, 41)
Legal Sex					
Female[2]	12 (60%)	6 (60%)	3 (60%)	3 (60%)	2 (50%)
Male[2]	8 (40%)	4 (40%)	2 (40%)	2 (40%)	2 (50%)
Gender[3]					
Female[2]	9 (45%)	5 (50%)	2 (40%)	2 (40%)	1 (25%)
Male[2]	6 (30%)	3 (30%)	2 (40%)	1 (20%)	2 (50%)
Other[2]	1 (5%)	0	1 (20%)	0	0
N/A[2]	4 (20%)	2 (20%)	0	2 (40%)	1 (25%)

[1]Median (Range); [2]n (%);[3]self-reported. DL= dose level. N/A=not reported

Supplementary Table 2. JYNNEOS vaccinated volunteers' characteristics

Characteristic	JYNNEOS, N = 19
Age (years). Median (min-max)	36 (25-81)
Gender (n of tot):	
Female	2/19
Male	17/19
Days since vaccination. Median (min-max)	98 (37-153)
Vaccination route, 1st/2nd dose (n of tot):	
SC/SC	2/19
ID/ID	4/19
SC/ID	7/19
ID/SC	2/19
Other	4/19

SC= subcutaneous, ID= intradermal

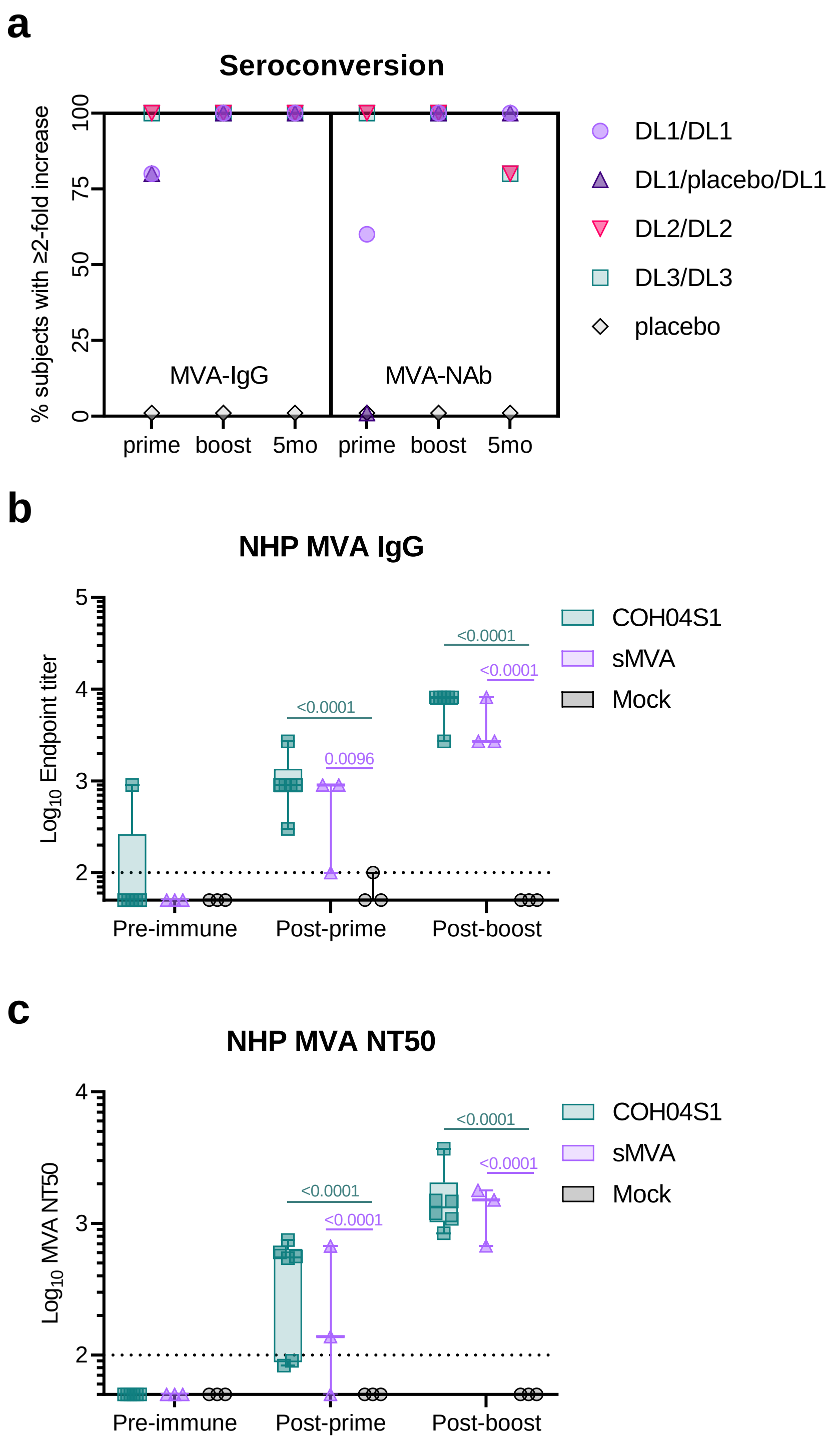

Supplementary Figure 1. Related to Figure 1. MVA-specific humoral response in COH04S1-vaccinated healthy adults and sMVA- and COH04S1-vaccinated NHP. a. Seroconversion rate. MVA-specific IgG endpoint titers measure by ELISA and MVA-specific neutralizing antibodies (NAb) measured using a MVA neutralization assay were measured in subjects before vaccination, post-prime vaccination, and at one- and five-months post-booster vaccination with

COH04S1 at dose-level (DL) 1 (DL1/DL1 and DL1/placebo/DL1), DL2 (DL2/DL2), and DL3 (DL3/DL3). Placebo controls were included. Shown is the percentage of seroconverted volunteers with a ≥2-fold increase in MVA-specific IgG titers and MVA NAb titers at different time points post-vaccination with COH04S1 compared to before vaccination. **b-c.** MVA-specific humoral responses in COH04S1- and sMVA-vaccinated NHP. NHP were two-times vaccinated with $2.5x10^8$ pfu (DL3) of COH04S1 (n=6) or sMVA (n=3). Mock-vaccinated NHP were used as controls (n=3). MVA-specific IgG endpoint titers (b) and MVA-specific NAb (c) were measured one month after the first dose (post-prime), and one month after the second dose (post-boost) by ELISA and neutralization assay, respectively. Dotted lines represent lower limits of detection. Box plots show 25th-75th percentiles, lines indicate medians, whiskers go from minimum to maximum values. Two-way ANOVA followed by Tukey's multiple comparison test was used following log transformation. P-values<0.05 are indicated.

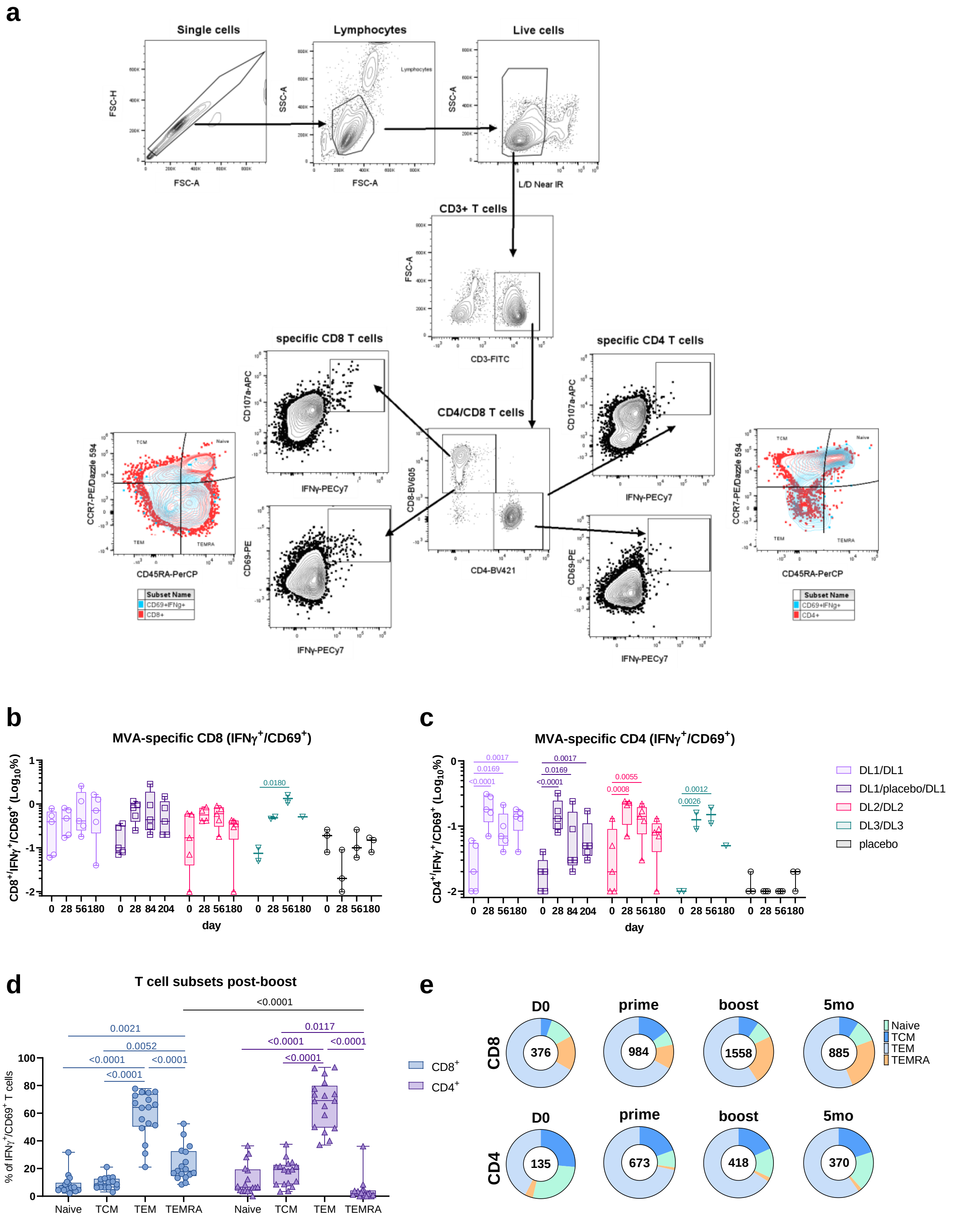

Supplementary Figure 2. Related to Figure 1. MVA-specific T cell response in COH04S1 vaccinees. a. Gating strategy. Shown is the gating strategy used to identify MVA-specific T cells. Gating was performed on single cells>lymphocytes>live cells>CD3^{+} cells> CD8^{+} or CD4^{+} cells>

CD107a^{+}/IFNγ^{+} or CD69^{+}/IFNγ^{+} cells. CD69^{+}/IFNγ^{+} double positive cells were used to identify CD8^{+} and CD4^{+} T cell memory subsets. Naïve cells were identified as CCR7^{+}/CD45RA^{+}; central memory (T_{CM}) cells were identified as CCR7^{+}/CD45RA^{-}; effector memory (T_{EM}) cells were identified as CCR7^{-}/CD45RA^{-}; and terminally differentiated effector memory (T_{EMRA}) cells were identified as CCR7^{-}/CD45RA^{+}. **b-c.** Activated MVA-specific T cells after vaccination with COH04S1. Percentages of MVA-specific IFNγ^{+}/CD69^{+} CD8^{+} (b) and CD4^{+} (c) T cells were measured in PBMC samples by cytofluorimetry at baseline, one-month after the first vaccination, and one- and five-months post-booster vaccinations with COH04S1 at dose-level (DL) 1 (DL1/DL1 and DL1/placebo/DL1), DL2 (DL2/DL2), and DL3 (DL3/DL3). Subjects who received placebo vaccination were used as negative controls. Only two DL3 volunteers had available PBMC samples for the analysis. 2-way ANOVA followed by Tukey's multiple comparison test was used following log transformation. P-values<0.05 are indicated. **d.** Phenotypic analysis of antigen-specific T lymphocytes was performed using samples collected one-month post-second dose. Shown are percentages of naïve, T_{CM}, T_{EM}, and T_{EMRA} cells measured in IFNγ+/CD69+ CD8+ and CD4+ T cell populations. In b-d box plots show 25th-75th percentiles, lines indicate medians, whiskers go from minimum to maximum values. 2-way ANOVA followed by Tukey's multiple comparison test was used to compare groups. P-values<0.05 are indicated. **e.** Relative contribution of activated CD8^{+} and CD4^{+} T cell memory subtypes in subjects vaccinated with COH04S1 at baseline, one-month after the first vaccination, and one- and five-months post-second dose. Total indicates the average number of IFNγ+/CD69+ CD8+ or CD4+ T cells/100 μl of blood.

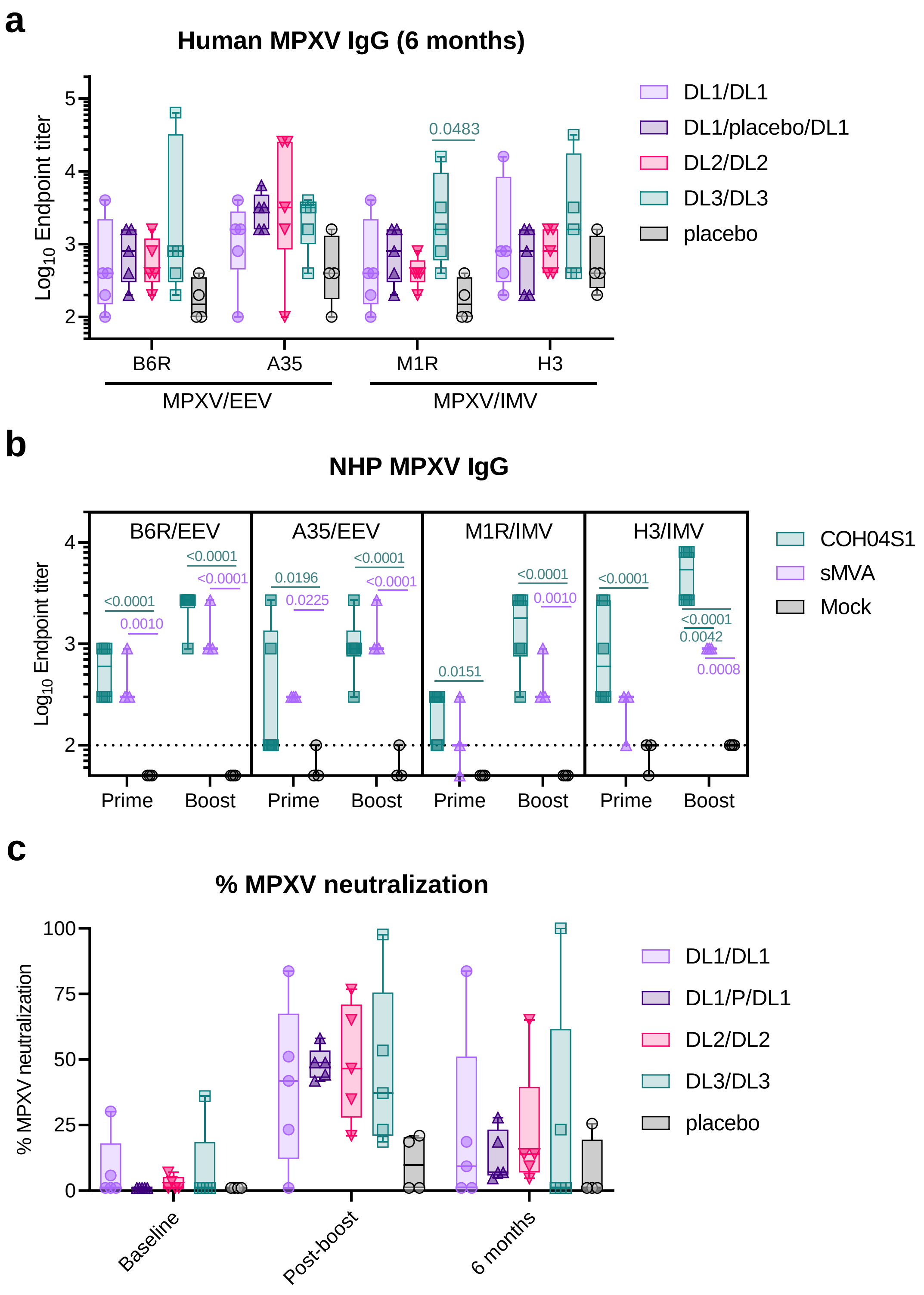

Supplementary Figure 3. Related to Figure 2. MPXV cross-reactive immunity in COH04S1 vaccinees and COH04S1- and sMVA-vaccinated NHP. a. MPXV-specific IgG. IgG endpoint titers to MPXV virion proteins B6R, A35, M1R, and H3 were measured in healthy adult six months

post-vaccination with COH04S1 at dose-level (DL) 1 (DL1/DL1 and DL1/placebo/DL1), DL2 (DL2/DL2), and DL3 (DL3/DL3) by ELISA. Placebo controls were included. P values <0.05 are indicated. **b.** MPXV-specific IgG in COH04S1- and sMVA-vaccinated NHP. NHP were two-times vaccinated with 2.5×10^8 pfu (DL3) of COH04S1 (n=6) or sMVA (n=3). Mock-vaccinated NHP were used as controls (n=3). MPXV-specific IgG endpoint titers to MPXV B6R, A35, M1R, and H3 proteins were measured one month after the first dose (prime), and one month after the second dose (boost) by ELISA. Dotted line represents lower limit of detection. **c.** MPXV neutralization**.** Percentage of MPXV neutralization was measured at the indicated timepoints in serially diluted serum samples of subjects vaccinated with COH04S1 at DL1, DL2, and DL3 by MPXV PRNT assay. Placebo controls were included. Box plots show 25th-75th percentiles, lines indicate medians, whiskers go from minimum to maximum values. In a-c 2-way ANOVA followed by Tukey's multiple comparison test was used to compare groups. P-values<0.05 are indicated. IMV= intracellular mature virions, EEV= extracellular enveloped virions.

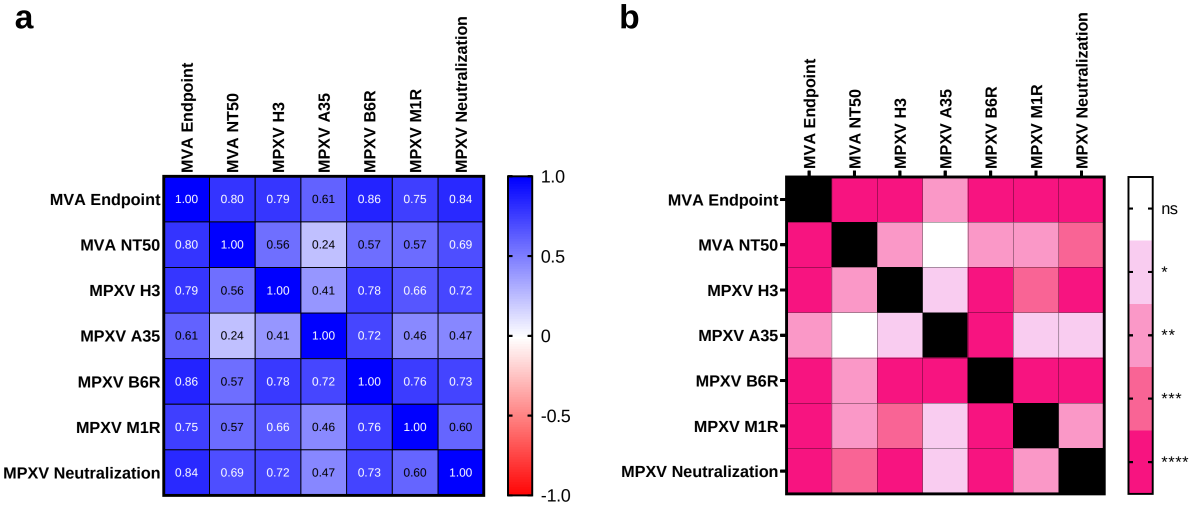

Supplementary Figure 4. Related to Figures 1-2. Correlative analysis of orthopoxvirus-specific and MPXV cross-reactive humoral responses in COH04S1-vaccinated subjects. Spearman correlation analysis was performed between the indicated MVA-specific and MPXV-specific humoral responses in COH04S1 vaccinated subjects one-month after the second dose. **a.** Spearman correlation coefficients were calculated and plotted as a matrix. **b.** Two-tailed p values were calculated and indicated as: ns= not significant (p>0.05), *=0.05 < p <0.01, **=0.01 < p < 0.001, ***=0.001 < p < 0.0001, ****=p < 0.0001.
