# Supplementary material for: Synthetic modified vaccinia Ankara vaccines confer cross-reactive and protective immunity against mpox virus
Source: Commun Med (Lond). 2024 Feb 16;4:19. doi: 10.1038/s43856-024-00443-9 (PMC10873322; doi:10.1038/s43856-024-00443-9)
Supplement: Supplementary file 3 — Description of Additional Supplementary Files [file 43856_2024_443_MOESM3_ESM.pdf]

Description of Additional Supplementary Files

File Name: Supplementary Data 1
Description: Source data for Figures 1-3 and Supplementary Figures 1-4
