# Supplementary material for: Synthetic modified vaccinia Ankara vaccines confer cross-reactive and protective immunity against mpox virus
Source: Commun Med (Lond). 2024 Feb 16;4:19. doi: 10.1038/s43856-024-00443-9 (PMC10873322; doi:10.1038/s43856-024-00443-9)
Supplement: Supplementary file 5 — Reporting Summary [file 43856_2024_443_MOESM5_ESM.pdf]

nature portfolio

Corresponding author(s): Flavia Chiuppesi

Last updated by author(s): Oct 4, 2023

Reporting Summary

Nature Portfolio wishes to improve the reproducibility of the work that we publish. This form provides structure for consistency and transparency in reporting. For further information on Nature Portfolio policies, see our Editorial Policies and the Editorial Policy Checklist.

Statistics

For all statistical analyses, confirm that the following items are present in the figure legend, table legend, main text, or Methods section.

n/a	Confirmed	
☐	☒	The exact sample size (*n*) for each experimental group/condition, given as a discrete number and unit of measurement
☐	☒	A statement on whether measurements were taken from distinct samples or whether the same sample was measured repeatedly
☐	☒	The statistical test(s) used AND whether they are one- or two-sided<br>*Only common tests should be described solely by name; describe more complex techniques in the Methods section.*
☒	☐	A description of all covariates tested
☐	☒	A description of any assumptions or corrections, such as tests of normality and adjustment for multiple comparisons
☐	☒	A full description of the statistical parameters including central tendency (e.g. means) or other basic estimates (e.g. regression coefficient) AND variation (e.g. standard deviation) or associated estimates of uncertainty (e.g. confidence intervals)
☐	☒	For null hypothesis testing, the test statistic (e.g. *F*, *t*, *r*) with confidence intervals, effect sizes, degrees of freedom and *P* value noted<br>*Give P values as exact values whenever suitable.*
☒	☐	For Bayesian analysis, information on the choice of priors and Markov chain Monte Carlo settings
☒	☐	For hierarchical and complex designs, identification of the appropriate level for tests and full reporting of outcomes
☐	☒	Estimates of effect sizes (e.g. Cohen's *d*, Pearson's *r*), indicating how they were calculated

Our web collection on statistics for biologists contains articles on many of the points above.

Software and code

Policy information about availability of computer code

Data collection	Attune NxT cytometer (Thermofisher); FilterMax F3 (Molecular Devices)
Data analysis	GraphPad Prism 8.3.0; Office Excel (v2019); Image-Pro Premier (v9.2; Media Cybernetics); SoftMax Pro v7 (Molecular Devices)

For manuscripts utilizing custom algorithms or software that are central to the research but not yet described in published literature, software must be made available to editors and reviewers. We strongly encourage code deposition in a community repository (e.g. GitHub). See the Nature Portfolio guidelines for submitting code & software for further information.

Data

Policy information about availability of data

All manuscripts must include a data availability statement. This statement should provide the following information, where applicable:

- Accession codes, unique identifiers, or web links for publicly available datasets
- A description of any restrictions on data availability
- For clinical datasets or third party data, please ensure that the statement adheres to our policy

All data generated or analyzed during this study are included in this published article, Supplementary Information, and Supplementary Data 1 file. All other data are available from the corresponding author on reasonable request.

Human research participants

Policy information about studies involving human research participants and Sex and Gender in Research.

Reporting on sex and gender	Subjects' legal sex and self-reported gender identity were collected. Data are summarized on Supplementary Tables 1 and 2. Out of 20 subjects vaccinated with COH04S1, 60% were female and 40% were male. Out of the 4 placebo recipients, 50% were female and 50% were male. Out of the 19 volunteers vaccinated with JYNNEOS, 2 were female and 17 were male.
Population characteristics	COH04S1 cohort: Healthy volunteers >18 and <55 year old, without previous SARS-CoV-2 infection, with BMI>18 or <35, with no underlying health conditions, and that had not received a poxvirus vaccination within a six-months period.<br>JYNNEOS cohort: Healthy volunteers, no age restriction. Fully vaccinated with JYNNEOS at least 30 days prior sample collection.
Recruitment	Study volunteers were recruited in the Los Angeles (COH04S1 cohort) and San Diego (JYNNEOS cohort) area, therefore the study population was representative of the local diversity.
Ethics oversight	City of Hope IRB#20447 was approved by an external Institutional Review Board (Advarra IRB). Plasma samples from Jynneos vaccinated volunteers were collected under IRB approval (VD-259) at the La Jolla Institute for immunology.

Note that full information on the approval of the study protocol must also be provided in the manuscript.

Field-specific reporting

Please select the one below that is the best fit for your research. If you are not sure, read the appropriate sections before making your selection.

[x] Life sciences [ ] Behavioural & social sciences [ ] Ecological, evolutionary & environmental sciences

For a reference copy of the document with all sections, see nature.com/documents/nr-reporting-summary-flat.pdf

Life sciences study design

All studies must disclose on these points even when the disclosure is negative.

Sample size	No sample size calculation was preformed.<br>For NHP study, n=6 COH04S1-vaccinated NHP, n=3 sMVA-vaccinated NHP and n=3 mock-vaccinated NHP.<br>64 CAST/EiJ mice were assigned to groups. 9 mice/group in experimental vaccine groups, 10 mice in control group. Sample size was based on: DOI: 10.1101/2022.12.17.520886 and DOI: 10.1073/pnas.2220415120.
Data exclusions	No data was excluded from the analysis. One mouse was found dead post-prime vaccination and one post-boost therefore data from these mice is not available. Death was determined to be not-vaccine related.
Replication	Serum samples were assayed in duplicates. Due to limitation in PBMC samples, T cell analysis was not performed in duplicates. TCID50 assay on lung samples was performed on quadruplicates.
Randomization	NHP were randomized by weight and/or sex to the experimental groups.<br>CAST/EiJ mice were randomly assigned to experimental or control groups.
Blinding	Samples were blinded to the investigators performing the assays. Investigators performing data analysis were not blinded to the groups.<br>Investigators performing animal vaccination were blinded to the groups.

Reporting for specific materials, systems and methods

We require information from authors about some types of materials, experimental systems and methods used in many studies. Here, indicate whether each material, system or method listed is relevant to your study. If you are not sure if a list item applies to your research, read the appropriate section before selecting a response.

Materials & experimental systems

n/a	Involved in the study
☐	☒ Antibodies
☐	☒ Eukaryotic cell lines
☒	☐ Palaeontology and archaeology
☐	☒ Animals and other organisms
☐	☒ Clinical data
☒	☐ Dual use research of concern

Methods

n/a	Involved in the study
☒	☐ ChIP-seq
☐	☒ Flow cytometry
☒	☐ MRI-based neuroimaging

Antibodies

Antibodies used

anti-human IgG HRP secondary antibody (BioRad 204005), used 1:3,000.
anti-monkey IgG(H+L) HRP secondary antibody (Thermo Fisher PA1-84631), used 1:10,000.
anti-CD3-FITC (Biolegend 300440), used 1:40
anti-CD4-BV421 (BD 566703), used 1:50
anti-CD8-BV605 (Biolegend 301040), used 1:100
anti-CD69-PE (BD 555531), used1:10
anti-CCR7-PE/Dazzle 594 (Biolegend 353236), used 1:20
anti-CD45RA-PerCP (Biolegend 304062), used 1:20

Validation

From the Biolegend website: Specificity testing of 1-3 target cell types with either single- or multi-color analysis (including positive and negative cell types). Each lot product is validated by QC testing with a series of titration dilutions.
From BD website: BD provides Quality Certificates for products certifying that their products have been manufactured and tested in accordance with BD specifications.
Antibodies were titrated in house for optimal performance.

Eukaryotic cell lines

Policy information about cell lines and Sex and Gender in Research

Cell line source(s)

ARPE-19 cells, ATCC CRL-2302. Human Retinal pigment epithelial (RPE) cell line. Male.
Vero E6 cells (ATCC, CRL-1586). African green monkey epithelial kidney cell line.

Authentication

Autenticated at the source.

Mycoplasma contamination

Tested for mycoplasma at the source.

Commonly misidentified lines
(See ICLAC register)

None.

Animals and other research organisms

Policy information about studies involving animals; ARRIVE guidelines recommended for reporting animal research, and Sex and Gender in Research

Laboratory animals

1 male and 11 female research naive, adult, African green monkeys, 3-7Kg purchased from a Bioqual's approved vendor.
63 male CAST/EiJ mice purchased from the Jackson laboratory.

Wild animals

No wild animals were used in the study.

Reporting on sex

COH04S1 group had 5 female and 1 male NHP. sMVA group had 3 female NHP, Mock-vaccinated group had 3 female NHP. Due to limitations in the number of male NHP it was not possible to balance the sexes in this study. CAST/EiJ mice were all male.

Field-collected samples

The study did not involve field-collected samples.

Ethics oversight

The NHP study was approved by: Bioqual (protocol 20-120) and and City of Hope (protocol 20075) Institutional Animal Care and Use Committees (IACUC). The mouse study was approved by: Bioqual (protocol 23-010) and and City of Hope (protocol 23014) Institutional Animal Care and Use Committees (IACUC).

Note that full information on the approval of the study protocol must also be provided in the manuscript.

Clinical data

Policy information about clinical studies
All manuscripts should comply with the ICMJE guidelines for publication of clinical research and a completed CONSORT checklist must be included with all submissions.

Clinical trial registration

This study is a retrospective evaluation of orthopoxviral-specific responses in a subgroup of 20 volunteers enrolled in the phase 1

Clinical trial registration	clinical trial aimed at testing safety and immunogenicity of COH04S1 at different dose levels (ClinicalTrials.gov, NCT046339466). Plasma samples from Jynneos vaccinated volunteers were collected as part of an observational study carried out at the La Jolla Institute for immunology (protocol VD-259).
Study protocol	Trial protocol can be accessed at: Chiuppesi et al., The Lancet Microbe, Volume 3, Issue 4, April 2022, Pages e252-e264. https://doi.org/10.1016/S2666-5247(22)00027-1
Data collection	COH04S1 cohort: Clinical data were collected between Dec 13, 2020, and May 24, 2021. JYNNEOS cohort: samples were collected between October and February 2023.
Outcomes	The primary objective was to evaluate the safety and tolerability of COH04S1 vaccine in healthy adults at 1·0×10^7 PFU, 1·0×10^8 PFU, and 2·5×10^8 PFU.

Flow Cytometry

Plots

Confirm that:

- [x] The axis labels state the marker and fluorochrome used (e.g. CD4-FITC).
- [x] The axis scales are clearly visible. Include numbers along axes only for bottom left plot of group (a 'group' is an analysis of identical markers).
- [x] All plots are contour plots with outliers or pseudocolor plots.
- [x] A numerical value for number of cells or percentage (with statistics) is provided.

Methodology

Sample preparation	PBMC samples were thawed and washed with RPMI media, then cells were counted and cultured with MVA, PHA or with media only for 24 hours. After this time, cells were stained with the superficial antibodies diluted in PBS for 15 minutes at room temperature. Then cells were permeabilized and fixed with Fix-Perm (BD) following the manufacture's instructions. After washing, cells were intracellularly stained for 30 min at 4°C, washed and resuspended in FACS buffer until acquisition.
Instrument	Attune NxT cytometer (ThermoFisher) with four lasers (405nm, 488nm, 561nm and 640nm)
Software	FlowJo 10.8.0 (BD) using a portal ID
Cell population abundance	The analysis of the populations was performed from the total cultured PBMC samples, cells were not sorted.
Gating strategy	The analysis strategy was performed by selecting the single cells first using the FSC-H Vs FSC-A parameters. Then the lymphocyte population was selected using the SSC-A Vs FSC-A parameters, monocytes were excluded by size. From lymphocytes, live cells were selected followed by CD3+ cells. Then CD4+ and CD8+ cells were selected and from each population, the specific T cells were determined as IFNg+/CD69+ or IFNg+/CD107a+ double positive cells considering the non-stimulated cells and FMO controls. Finally, the memory/naive (CCCR7 Vs CD45RA plots) cells were analyzed from the specific T cells considering the FMO controls.

- [x] Tick this box to confirm that a figure exemplifying the gating strategy is provided in the Supplementary Information.
